# The Effect of Language Dominance on the Selective Attention of Segments and Tones in Urdu-Cantonese Speakers

**DOI:** 10.3389/fpsyg.2021.710713

**Published:** 2021-07-30

**Authors:** Yi Liu, Jinghong Ning

**Affiliations:** ^1^Department of Chinese and Bilingual Studies, Hong Kong Polytechnic University, Hong Kong, SAR China; ^2^PolyU-PekingU Research Centre on Chinese Linguistics, Hong Kong, SAR China

**Keywords:** attention distribution, Cantonese tones, segments, bilingual, language dominance

## Abstract

To perceive a second language (L2), non-native speakers not only have to focus on phonological, lexical, and grammatical knowledge, but also need to develop a good mastery of L2 strategic knowledge, including selective attention and language planning. Previous research has found that non-tonal speakers are overtly attentive to segments, while tonal language speakers give more attention to tones. However, it is unclear how different dominant language speakers distribute their attention while processing segments or tones and segments and tones stimuli in non-native speeches. The present study also aims to examine the roles of language dominance play in the designed perceptual tasks. In the current study 20 Cantonese native speakers, 18 Cantonese-dominants, and 18 Urdu-dominants participated in an attention distribution experiment in Cantonese. The results show that the Urdu-dominants retain their L1 attentional strategy in the processing of Cantonese stimuli, classifying the stimuli along segments, while the Cantonese native speakers are more attentive to tones. Moreover, the Cantonese-dominants show a perceptual flexibility as highly proficient and experienced listeners. The results reveal that language dominance plays a vital role in listeners' attention distribution. The research also supports PAM-L2 theory on bilingual. The findings of the current study can be applied to Chinese language learning and teaching and language acquisition studies.

## Introduction

From a psycholinguistic perspective, language perception is the process of selecting, organizing, and interpreting information. Selective attention refers to a sensory skill in a cognitive process where listeners make a selection of certain sub-syllabic dimensions while ignoring the irrelevant information (Treisman, 1964). To perceive L2 sounds, listeners not only need a large store of L2 language knowledge (e.g., phonology, lexicon, grammar), but also have to master L2 strategic knowledge, including selective attention and language planning (Treffers-Daller, 2019). Recently, there has been an increasing interest in the way tonal and non-tonal speakers distribute their selective attention toward segment and tone (Braun and Johnson, 2011; Zou et al., 2017). When perceiving a speech sound, tonal speakers pay simultaneous attention to both segmental and tonal dimensions, as they tend to use pitch information as the primary cue in lexical and sentential meaning (Zou et al., 2017). When processing a non-native tonal language, non-tonal speakers may find it hard to give attention to tone due to the absence of a sensitivity toward tone (Braun and Johnson, 2011; Zou et al., 2017). Moreover, it has been reported that tonal sensitivity is expected to be gradually acquired by non-tonal speakers as tonal L2 experiences and proficiency improve (White et al., 2017; Zou et al., 2017). But it is still unclear how sensitively and automatically non-tonal speakers will be able to allocate their attention to a tonal L2, even when they have developed into highly proficient and fluent target language users. As an extension of Perceptual Assimilation Model (PAM, Best, 1995), PAM-L2 (Best and Tyler, 2007) proposed that non-native language learners may assimilate L2 sounds into their L1 categories, or establish new categories for the unassimilated L2 contrasts. The current study adopts the framework of selective attention and PAM-L2 to unveil the attention distribution of different dominant language speakers on perceiving Cantonese segments and tones.

According to Grosjean (1998), bilinguals were the population who use more than (include) two languages in their everyday lives. The two languages of bilingual speakers are usually imbalanced, with the language more frequently used serving as a base language, and called a stronger or a dominant language, while the other one becomes a weaker language (Pavlenko, 2014; Treffers-Daller, 2019). This suggests that bilinguals do not generally have exactly the same competencies or skills in their native and target languages. More researchers see bilingual speakers as unique and configured language users, rather than a population of two monolingual speakers (Sebastián-Gallés and Soto-Faraco, 1999; Pallier et al., 2001; Nicoladis, 2006).

The current study examines how bilingual listeners accommodate language systems, when distributing their attention between non-native segments and tones. Since previous studies have focused on how bilingual speakers acquire L2 phonetic knowledge, the current study will provide more evidence on language-specific selective attention of bilingual speakers when processing non-native segments and suprasegments. Moreover, the present research investigates the role that language dominance plays in the bilingual speakers' attention distribution of L2 tones and segments. Tonal Cantonese native speakers and bilinguals whose L1 is the non-tonal language of Urdu were employed to participate in the Cantonese attention distribution tasks. The bilinguals were middle-school students, who were exposed to Urdu first and started learning Cantonese between the ages of two to thirteen, and were dominant in either Cantonese or Urdu. In the current study, Urdu was L1 for these bilinguals, and Cantonese was considered as L2.

The significance of the current study is to reveal Cantonese-dominants and Urdu-dominants' attention allocation when they process Cantonese segments and tones, and further discuss the role that language dominance plays in the perceiving of non-native sounds. The observation obtained from the study can contribute to Chinese language learning and teaching and second or foreign language acquisition research.

## Literature Review

### Distribution of Attention to Segmental and Tonal Information

Based on the previous studies, the acquisition of Chinese involves various aspects (Ma et al., 2017; Gong et al., 2018, 2020a,b,c, 2021). Perceptual performance of new categories in non-native listeners incorporates the development of perceptual sensitivities to new acoustic dimensions (Goldstone, 1993). Lexical tone is a new acoustic dimension for non-tonal language speakers. In learning a tone language, non-tonal speakers not only have to develop phonological categories for tones, but must also redistribute their selective attention to both segmental and tonal dimensions (Zou et al., 2017). According to Strange and Shafer (2008) and Strange (2011), speech perception is a “purposeful and information-seeking” activity, where adult listeners can use a “highly over-learned” and highly automatic program (the selective perceptual routine), referring to their L1 systems. With the assistance of such a selective perceptual routine, listeners can automatically extract enough information through various linguistic conditions. According to Treffers-Daller (2019), the selective perceptual routine proposed by Strange and other scholars can be regarded as one type of strategical knowledge. As one of the executive functions, Selective attention influences listeners' perceptions at a higher-ordered and abstract level (Diamond, 2013). Strange and Shafer (2008) claimed that only native speakers could automatically utilize the selective perceptual routine, whereas late adult learners had only a slim chance of developing an exact L2-like selective perceptual routine. Strange (2011) posited that even when native speakers are exposed to “sub-optimal listening conditions” or distracted by another task, they can still phonologically discriminate different native phonetic contrasts in a rapid and robust way. For late adult learners, as some L2 phonetic contrasts do not occur in their L1, much greater cognitive resources are required to extract sufficient information in L2 perception (Strange, 2011).

Some neurocognitive and behavior studies on selective attention suggest that when more L2 proficiency is accumulated for non-native learners, they are able to access a more automatic attentional strategy, specific to L2 (Steinhauer, 2014; White et al., 2017; Zou et al., 2017). Francis and Nusbaum's (2002) study showed that after training, English listeners redistributed their attention to different perceptual dimensions for the establishment of new L2 phonetic categories, and were able to approximate the behavior of native Korean listeners in the post-test. Zou et al. (2017) invited native tonal Mandarin adult speakers, non-tonal Dutch speakers (who had never learned Mandarin), and Dutch-speaking learners of Mandarin to participate in an ABX task in which the target syllable in disyllabic non-words varied along tonal or segmental dimensions. Their results supported the findings of Braun and Johnson (2011), demonstrating that Mandarin speakers were attentive to both segmental and tonal information in the processing of Mandarin stimuli, whereas native Dutch speakers mainly depended on the segmental dimension. A developmental trajectory of L2-specific selective attention for learners was revealed, showing that beginners were more likely to ignore tonal information compared with advanced learners (Zou et al., 2017).

Prior studies on selective attention have focused on whether L2-specific attentional strategy could be acquired by less experienced learners or by adult learners (Strange, 2011). However, it is still unclear whether the learners are able to acquire an L2-like attentional strategy on achieving a high L2 proficiency and becoming bilingual.

### Research on Language Dominance

Snape and Kupisch (2016) defined the dominant language as the “more proficient” or “further developed” language for bilinguals. The dominant language of bilinguals can be either their L1 (Sebastián-Gallés et al., 2005; Amengual, 2016) or their L2 (Antoniou et al., 2012), whichever have been primarily and regularly utilized by language speakers in daily conversations. The two main underlying dimensions of the language dominance are language proficiency and language use (Luk and Bialystok, 2013; Treffers-Daller, 2019). Language proficiency shows how well languages are known, and language use illustrates how frequently bilinguals use their languages (Treffers-Daller, 2019).

Personal and experiential factors also play important roles in constructing listeners' language dominance. For instance, according to Piske et al. (2001), the age of onset learning (AOL) and the age of arrival in the target language-speaking area (AOA) correlate tightly in the performance of bilingual speakers, illustrating a cumulative exposure to L2 for bilinguals. Moreover, success in L2 acquisition also depends heavily on personal factors such as educational level (Hamann et al., 2018), and length of residence (LOR) in the target language-speaking area (Flege and Fletcher, 1992).

Previous studies have focused on the role that language dominance plays in the processing of L2 phonetic contrasts, such as vowels and consonants. It has been reported that bilinguals show strong bias toward their dominant language in speech perception tasks (Antoniou et al., 2012; Molnar et al., 2016). Molnar et al. (2016) assessed the non-linguistic tone grouping biases of Spanish monolinguals, and three groups of Basque-Spanish bilinguals with different levels of Basque experience. Participants' non-linguistic rhythm preferences were assessed in response to non-linguistic tones alternating in either intensity (intensity condition) or in duration (duration condition). In the intensity condition, all groups showed a trochaic grouping bias, as predicted by the iambic-trochaic law. The two other bilingual groups showed no significant bias. Overall, the results indicated that duration-based grouping mechanisms are biased toward the phrasal prosody of the native and dominant language.

Concerning how to define language dominance and classify participants in experimental task, an integrated perspective has been proposed. Birdsong et al. (2012) suggested that language dominance can be interpreted through dominance scores according to the questionnaire survey of the Bilingual Language Profile (BLP). The BLP allows us to access bilinguals' dominance on the following aspects: age of acquisition of L1 and L2 (language history); frequency and context of use (language use); competence in different skills (language proficiency), and attitudes toward each language (language attitudes). These factors are organized into four modules with equal weightings. The BLP method has been widely introduced in bilingual studies and in empirical and laboratorial linguistic studies (e.g., Amengual, 2016).

Views vary as to how bilinguals accommodate their weaker and stronger languages. The “one-activation” view suggests that speakers' weaker and stronger languages are separately activated, without interfering with each other (Amengual, 2016; Blanco et al., 2016). Amengual (2016) investigated the perception and processing of mid-vowel contrasts in Majorcan Catalan by early Spanish-Catalan bilinguals. Participants were required to identify the target vowel in a binary forced choice identification task, and to discriminate between vowel pairs in an AX discrimination task. In the third experiment, those bilingual speakers were asked to distinguish words and non-words, encoding the target vowels, from a large stimuli pool. The result showed that early Spanish-Catalan bilinguals in Majorca could categorically perceive the Catalan vowels in a native-like way. And the bilinguals had great difficulty distinguishing between words and non-words that differed in the Catalan vowel contrasts.

In contrast, the “co-activation” view suggests that bilinguals show simultaneous activation of both languages even when processing only one (Nicoladis, 2006). According to a longitudinal study of French-Swedish bilingual children, Schlyter (1993) found that the stronger language was well-developed by non-native speakers, whereas the weaker language would be incompletely acquired. Some bilingual studies referred to this “incomplete acquisition” as “interference, transfer, or crosslinguistic influence” (see in Grosjean, 2012a). Grosjean (2012a) proposed that the weaker language is comparatively less activated compared to the strong language, and listeners may not completely inhibit the interference from the weaker language, especially in a bilingual language mode, where bilinguals are exposed to both strong and weaker languages.

Sebastián-Gallés and Soto-Faraco (1999) explored whether highly experienced early bilinguals, who have already mastered L2 categories, can perform as well as native speakers. Catalan-dominant Catalan/Spanish bilinguals as well as early Spanish-dominant Spanish/Catalan bilinguals (who were exposed to only Spanish or Catalan before the age of four and were proficient in both languages at the time), took part in the experiment. The results showed that Spanish-dominant bilinguals showed worse perceptual results than the group of Catalan-dominant bilinguals. This suggested that an early exposure to a new language is not sufficient to overcome the influence of L1 when perceiving L2 categories.

Antoniou et al. (2012) emphasizes the “flexible” role in language dominance of bilingual speakers in speech processing. Grosjean (1989) states that “a bilingual speaker is not two monolinguals in one,” and that bilinguals should be considered as an unique and configured population very different from a monolingual one (Antoniou et al., 2012). It is posited that such bilingual “flexibility” allows listeners to perform as a monolingual speaker or a bilingual speaker according to their tasks (Antoniou et al., 2012), or the language mode in which they are immersed (Grosjean, 2012b), and that bilingual speakers would perform differently in terms of different experimental tasks.

Language mode is the state of activation of the bilingual languages and language processing mechanisms at a given point in time (Grosjean, 2012b). The language mode framework (Grosjean, 1998, 2012b) illustrates that if only one language mode (e.g., L2) is provided in the experiment process, L1-related memories will not be activated (or only slightly) for early bilinguals, and they will perform exactly like a native speaker of L2; and vice versa when only L1 is provided. On the contrary, in a mixed language mode where both L1 and L2 are provided, listeners' weaker and stronger languages will be activated, but the weaker language will not be activated as strongly as the dominant one. In the mixed language mode, listeners are expected to perform as bilingual speakers. Listeners are able to shift their roles in different language modes, thus showing “flexibility” of bilingual speakers.

### PAM Family and Its Application to Bilingualism

In the domain of second language acquisition, PAM has been proposed in accounting for L2 users' perception of speech segments. Perceptual Assimilation Model proposes that language learners are likely to refer to their L1 phonology system when discriminating between L2 phonetic contrasts, and to make a perceptual assimilation between the two phonology systems (Best, 1995). The Perceptual Assimilation Model for Suprasegmentals (PAM-S) suggests that language learners tend to assimilate L2 prosodic contrasts to L1 prosodic categories (So and Best, 2011).

An extension to L2 perceptual learning, PAM-L2 (Best and Tyler, 2007) predicts that non-native listeners may assimilate L2 contrasts into L1 categories, or establish new categories for the unassimilated L2 sounds. Antoniou et al. (2012) attempted to extend the L2 acquisition models to account for the case of bilingual speakers, proposing that L1 and L2 systems are both well-developed, but it is not excluded that there exist a L1/L2 overlap, within which some phonetic properties are shared between L1 and L2. In other words, for early bilinguals, L1 can affect L2 since both the L1 and L2 can be activated in the common L1/L2 overlap. In Antoniou et al. (2012), the L2-dominant bilinguals whose L1 was Greek and L2 was English categorized, rated, and discriminated stop-voicing in both English and Greek. The results showed that the bilinguals biased to their dominant language when distinguishing phonetic contrasts, while they were influenced significantly by the language mode when making goodness-of-fit ratings between L1 and L2 phonetic categories. The bilinguals in Antoniou et al. (2012) thus showed flexibility to perform like monolinguals in the discrimination task and behave in a bilingual-like way in the categorization task.

Antoniou et al. (2012) examined the effectiveness of PAM-L2 in predicting the perceptual performance by bilingual speakers on a segmental level. The current study focuses on tones and selective attention to attest whether PAM-L2 theory and Antoniou et al.'s observations on bilinguals can predict how the Urdu-dominant and Cantonese-dominant Urdu/Cantonese bilinguals distribute their selective attention to segments and tones when processing L2 speech.

### Current Study

In relation to how bilingual speakers accommodate their stronger and weaker languages, previous findings that listeners' L1 phonology did not influence the perception of L2 consonants or vowels (Amengual, 2016; Blanco et al., 2016) evidenced the view of “one-activation.” Other researchers supported the view of “co-activation” by reporting L1 phonology interference when perceiving L2 segment contrasts (Schlyter, 1993; Sebastián-Gallés and Soto-Faraco, 1999; Pallier et al., 2001; Nicoladis, 2006). The viewpoint of “flexibility,” much less discussed by previous studies than the former two views, focuses on the flexible and shifting roles of L1 and L2 when bilinguals are processing speech in different tasks (Antoniou et al., 2012) and language modes (Grosjean, 2012b). As most research has mainly focused on the interference of phonology, it remains unclear how L1 and L2 are activated when bilinguals are processing cognitively demanding perception tasks in which language-specific attentional strategies are needed. To look more closely at this issue, three research questions are addressed: (1) How do language dominance speakers allocate their selective attention to segmental or tonal dimensions when processing L2 contrasts? (2) How do language dominance speakers distribute their selective attention to segmental and tonal dimensions while processing L2 stimuli? (3) What is the role that language dominance plays in the perceptual process?

Native Cantonese speakers, Cantonese-dominant and Urdu-dominant bilingual speakers are invited to undertake a revised ABX task from Zou et al. (2017). The bilingual speakers are immigrants to Hong Kong, with Urdu as L1 and Cantonese as L2. In the task the subjects are required to identify whether target X sounds closer to the preceding stimulus A or B, which has the same segment and/or tone as X. In the task of segment-*and*-tone, listeners are provided with an accurate segment and tone in A or B. In the task of segment-*or*-tone, A or B contains only one correct dimension of segment or tone. For example, an accurate tone appears in A, and an accurate segment appears in B. The task of segment-or-tone allows the listeners to choose only one dimension, being forced to neglect the other. Two pairs of CVCV nonce words were stimuli for the perceptual tasks. On the initial syllables, Cantonese Tone 2 (low-rising) or Tone 4 (low-falling) were carried by the syllable, and the second syllable for each disyllabic nonce word was neutralized as the Cantonese high-level tone. The second syllable for each disyllabic nonce word was neutralized as Cantonese high-level tones (Tone 1), which is the most stable tone in Cantonese that can facilitate the discrimination of the preceding or the following tones (Qin and Mok, 2011). Comparison of the results of the two tasks will enable examination of how bilingual and Cantonese native speakers distribute their attention between tonal and segmental dimensions.

As illustrated by Strange and Shafer (2008) and Strange (2011), native tonal speakers are able to distribute their limited attention to both tonal and segmental dimensions, automatically driven by their native attentional strategy. Whereas, the way tonal speakers distribute their selective attention is distinct from that of Urdu speakers (non-tonal). For Urdu native speakers, who only depend on the segmental dimension, the tonal dimension would appear to be a new L2 category for them. Thus, it is predicted that where only a L2-specific attentional strategy is allowed, Urdu-Cantonese bilinguals might retain their L1 (Urdu) attentional strategy and rely heavily on segmental dimension, even though they are processing L2 (Cantonese) speeches. In addition, a phonological influence may co-occur with such L1 attentional interference. according to PAM-S, bilingual speakers may assimilate the high-rising Cantonese tone as Urdu question intonation, since they share rising pitch contours (So and Best, 2011), while the Cantonese low-falling tone may be assimilated as Urdu statement intonation due to the overall descending pitch tendency in a statement sentence (So and Best, 2011). Therefore, discrimination difficulty is predicted to be relatively low. If the phonology impact exists for bilingual speakers, it will facilitate the L2 processing, rather than impede it in the process. Hence, if the bilinguals classify L2 contrasts overtly along segments, it is predicted that this will be interfered by their L1 attentional strategy, which supports “co-activation.” If the bilinguals automatically adopt a L2-like strategy and focus more on tones, they could be considered as Cantonese monolinguals under a Cantonese mode, which supports “one-activation.” Furthermore, there is a possibility that the bilinguals will behave like Cantonese natives in the task of “segment-and-tone,” whilst performing like unique L1–L2 bilinguals in the task of “segment-or-tone.” This is because the latter task has a low memory demand, while the former one requires listeners to use comparatively more cognitive resources to make responses. If so, the results will suggest that bilingual speakers are able to flexibly shift their roles according to task conditions. This would also support Antoniou et al.'s (2012) claim on PAM-L2, positing that if an independent L2 system is fully established for bilinguals, L1 and L2 still interfere with each other in a language overlap. Since Antoniou et al. (2012) attested PAM-L2 on a segmental level, the current study will provide more evidence from the perspective of the higher-ordered executive level and the suprasegmental level.

## Methods

### Participants

Taking part in the experiment were 36 bilingual speakers with Urdu as L1 and 20 native Cantonese speakers (10 female, 10 male). Both the bilingual participants (mean age = 12.1 years, *SD* = 3.2) and native Cantonese speakers (mean age = 13.3 years, *SD* = 2.1) were year one students, studying in local Hong Kong secondary schools. According to the self-reports, in the first 1 or 2 years of their lives, the bilingual participants had been exposed only to Urdu. They spoke Urdu at home and the medium of instruction for school classes was Cantonese. All the participants were healthy, right-handed and did not suffer from any hearing difficulties.

Each participant was required to complete the BLP questionnaire (Birdsong et al., 2012), provided in either Urdu or Cantonese, depending on participant preference. The BLP is an instrument for assessing language dominance, and includes the following four modules: subjects' language history; language use; language proficiency, and language attitude. We made revisions to BLP questionnaires designed for adult bilingual speakers, to be more suitable to middle school aged bilinguals. For example, we substituted the “work” language domain to a “school” occasion, a more common environment for middle-school students.

The participants were further classified as Urdu-dominant or Cantonese-dominant based on their self-reporting of the BLP questionnaires, which generated Urdu and Cantonese particular scores for the four modules. And a global language dominance scores (LDSs) were generated for each bilingual speaker, with the Urdu score subtracted from the Cantonese score. According to the four modules of the BLP, the participants gave self-rating on a 20-point scale for language history, a 10-point scale for language use, and a 6-point scale for the other two modules. The coefficients were multiplied for each module score in order to weigh the four dimensions equally. This gave the sum of the four revised module scores for L1 and L2 in separation. The LDS were then calculated by subtracting the total scores of L1 from L2 for each bilingual speaker.

Participants with negative scores were classified as Urdu/L2 dominant, while participants with positive scores were classified as Cantonese/L2 dominant. Eighteen Urdu-dominants (11 female and 7 male) and 18 Cantonese-dominants (10 female and 8 male) were selected as participants in the experiment. The 36 bilingual participants emigrated to Hong Kong between the ages of one and ten, and commenced their Cantonese learning between the ages of two and thirteen. In comparison with the Urdu-dominant speakers, the Cantonese-dominant subjects had a much lower AOA and AOL, and a significantly longer LOR (illustrated in Table 1). Also, the Cantonese-dominant speakers used Cantonese far more frequently than the Urdu-dominants did on most occasions (in class, and after class, etc.), regarding the Cantonese to Urdu ratio of language use (see Table 1).

**Table 1:** Individual information (mean values and ranges) of AOA, AOL, LOR, and the Cantonese to Urdu ratio of language use for bilinguals in terms of different occasions (home, school, others).

	**Age-related information (in years)**			**The Cantonese to Urdu ratio of language use**		
	**AOA**	**AOL**	**LOR**	**In the class class**	**After class**	**Others**
Cantonese-dominants	4.3	4.9	11.3	3.1	1.9	2.1
	*1–6*	*2–6*	*9–15*	*1.2–4.0*	*0.9–2.3*	*1.3–2.9*
Urdu-dominants	7.7	8.1	4.4	0.9	0.7	1.1
	*5–10*	*5–13*	*2–6*	*0.5–1.2*	*0.4–1.0*	*0.5–1.3*
*T-pair (in 2 tails)*						
*t*	35.2	40.2	54.6	67.5	44.5	47.8
*p*	<0.001	<0.001	<0.001	<0.001	<0.001	<0.001

*T-pair test (in two tails) results are shown at the bottom of the table to compare the differences of the variables between the two language dominant groups*.

**Figure 1:**
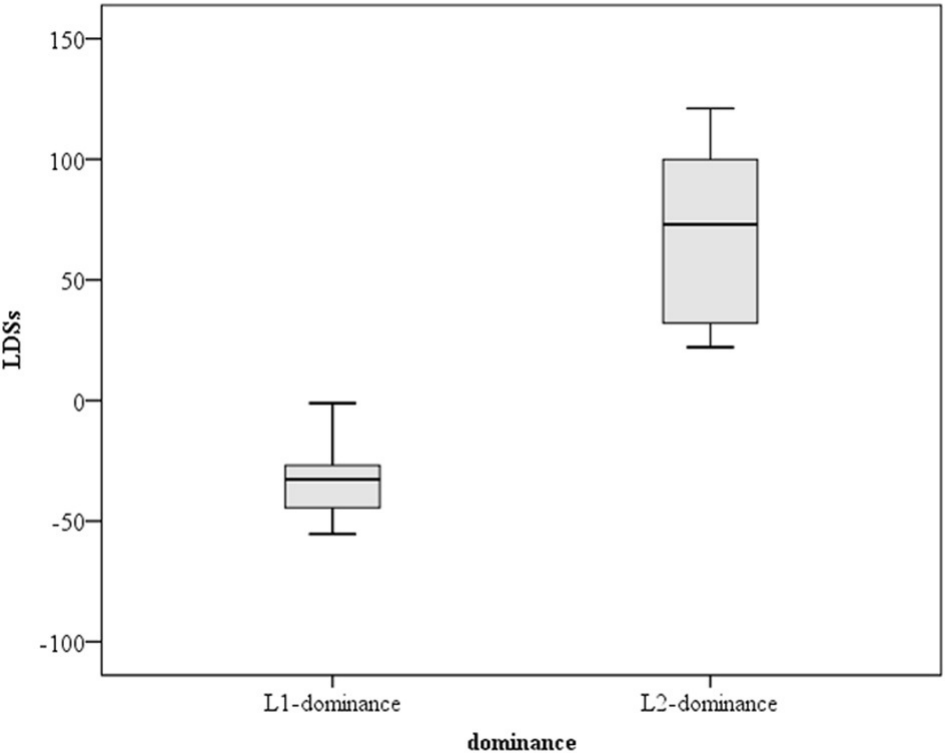
The distribution of language dominance scores according to the Bilingual Language Profile.

Language dominance scores ranged largely from −55.4 (strongly L1 dominant) to 121 (strongly L2 dominant), illustrating that the subjects exhibited different degrees of language dominance. Hence, it is interesting to examine how the non-native learners with different overall LDSs showed variances in their ABX performances. Figure 1 illustrates the distribution of LDSs of the bilingual speakers.

### Stimuli

Revised from the experimental materials in Zou et al. (2017), who studied listeners' attention distribution and integration of Mandarin segments and tones, two pairs of CVCV nonce words /kasu/-/tafu/ and /biso/-/diso/ were selected to avoid the lexical interference in Cantonese. Two female and one male Cantonese native speakers recorded the disyllables with CoolEdit 2.0 on a Lenovo ThinkCentre desktop computer (i5 core, USB interface: 3.0) with a Boom microphone, in the audio booth at Hong Kong Polytechnic University. The speakers were shuffled in each ABX combination instead of it being produced by the same speaker, in order to increase phonetic variability and listeners' memory load (Zou et al., 2017). Roman script with Cantonese tone marks of the nonce words was provided to the speakers, who had been trained in the pronunciation and the Cantonese scripts of the nonce words. The native speakers were asked to produce the disyllabic pairs with an interval of around one second in a natural speaking speed and the files were sampled at 44,100 Hz.

**Figure 2:**
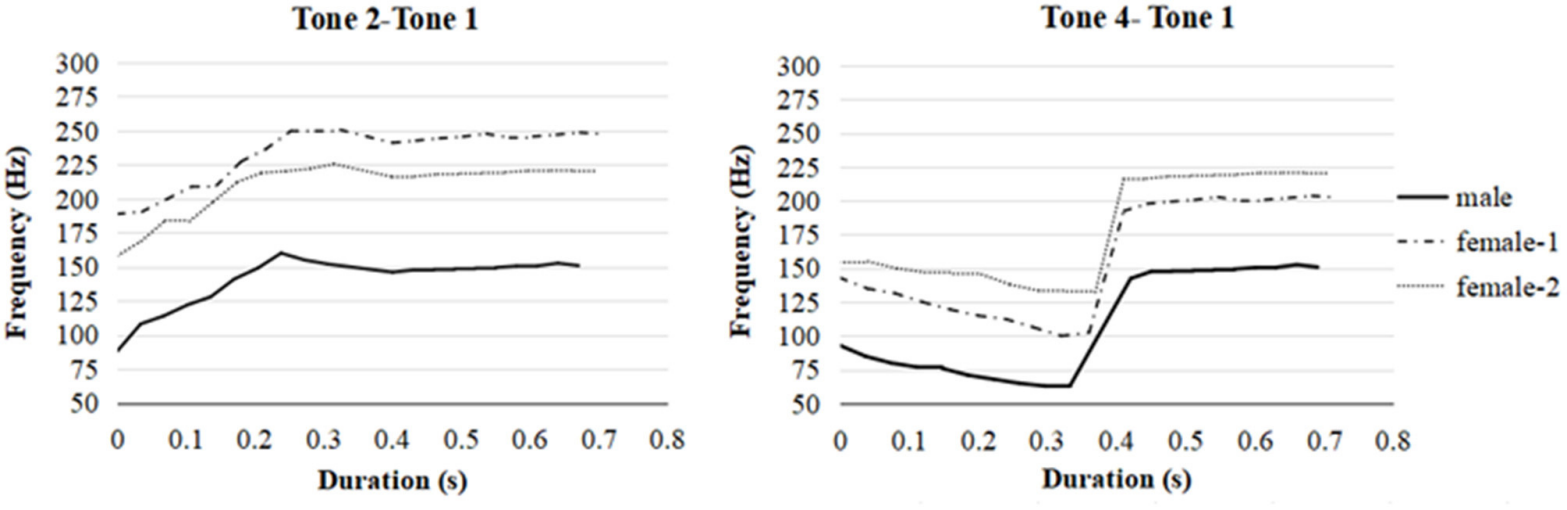
The pitch contours of disyllabic non-words produced by one male native speaker and two female native speakers in Cantonese. The pitch frequencies were averaged across /kasu/-/tafu/ and /biso/-/diso/.

The pitch contours, which were averaged across different disyllables for each speaker were depicted in Figure 2. The stimuli showed phonetic variability with the pitch range of the three speakers were distinct from each other (female 1: 100–250 Hz; female 2: 132–225 Hz; male: 63–155 Hz). The Tone 2 in the first syllable raised from a low point of the pitch scale to a much higher pitch for each native speaker (female 1: 188–250 Hz; female 2: 158–225 Hz; male: 88–155 Hz). The Tone 4 in the first syllable fell from a low pitch to a lower one, exhibiting a falling contour for each speaker (female 1: 143–100 Hz; female 2: 155–132 Hz; male: 93–63 Hz). The Tone 1 in the second syllable showed stable high pitch contours when it was preceded by Tone 2 (female 1: 241–248 Hz; female 2: 216–220 Hz; male: 146–152 Hz), or Tone 4 (female 1: 192–202 Hz; female 2: 216–220 Hz; male: 142–152 Hz). The pitch contours obtained in the tokens correspond with the description of Cantonese tones in Hao (2012), with tone transcription of 25, 21 and 55 for Tone 2, Tone 4, and Tone 1, respectively. The tone transcription suggested by Chao (1930) is a method to mark tone pitch values with 1 stands for the lowest pitch and 5 for the highest.

Two ABX tests were conducted with segment-and-tone and segment-or-tone conditions (see Zou et al., 2017). In the segment-and-tone task, participants were asked to decide whether target X matched either A or B. In the segment-or-tone task, target X matched either the segmental or tonal dimension with A or B. The nonce word pairs were arranged for each ABX task according to the following criteria: (1) the target X contained the same tone and/or segment as A or B, (2) the stimuli order could be ABX or BAX, and (3) the speakers were shuffled in each ABX combination instead of being produced by the same speaker, in order to increase phonetic variability and listeners' memory load. Thus, for each task, we got 16 ABX stimuli (two non-word pairs × two Cantonese tones × two AB orders × two matches with A or B). The arrangement of stimuli is illustrated in Table 2, which shows only one AB order.

**Table 2:** Arrangement of stimuli in ABX tasks.

**Condition**	**A**	**B**	**X**
Segment-and-tone	ka2su1	ta4fu1	ka2su1/ta4fu1
	ka4su1	ta2fu1	ka4su1/ta2fu1
	bi4so1	di2fo1	bi4so1/di2fo1
	bi2so1	di4fo1	bi2so1/di4fo1
Segment-or-tone	ka2su1	ta4fu1	ka4su1/ta2su1
	ka4su1	ta2fu1	ka2su1/ta4fu1
	bi4so1	di2fo1	di4fo1/bi2fo1
	bi2so1	di4fo1	di2fo1/bi4fo1

*The number represents a Cantonese tone mark; 1, 2, and 4 stand for Tone 1, Tone 2, and Tone 4 in Cantonese, respectively*.

### Procedure

The participants took part separately in the experiment in a quiet classroom in a local secondary school, with the Praat experiment script run in a computer (Lenovo ThinkCentre desktop, i5 core, USB interface: 3.0) for each participant. Before the start of the experiment, instructions were given by Cantonese native speakers. Participants were asked to listen to three nonce words (A, B, and X) and indicate if X sounded more similar to A or B by a mouse click on “1” or “2” shown on their computer screen, without any script shown. In each task there was a 600 ms interval between standard A and standard B, and X appeared after a 900 ms pause (Braun and Johnson, 2011). The inter-stimuli interval between the two tasks was 2,500 ms, and if the subject failed to respond within the interval, the stimulus would be shown again later on, to ensure no missing data in the experiment. The subjects had been given a 4-min familiarization task in the segment-and-tone condition before the formal experiment began. In the formal experiment, for each individual, there were five repetitions for each stimulus, resulting in 160 ABX tasks (16 ABX stimuli × 2 tasks × 5 repetitions) in total. The whole experiment was conducted within 30 min for each participant. The whole experiment was conducted for around 20 min for each participant.

## Data Analyses and Results

Reaction time and response rates were collected throughout the experiment. Response rate was calculated according to the percentage of “correct” (for segment-and-tone) or “segment” (for segment-or-tone) responses out of the five responses for each participant, and each ABX stimulus. For the native speakers group, we got 640 response rates (16 ABX stimuli × 2 tasks × 20 subjects) as well as 3,200 reaction times (16 ABX stimuli × 2 tasks × 20 subjects × 5 repetitions); 576 response rates (16 ABX stimuli × 2 tasks × 18 subjects), and 2,880 reaction times (16 ABX stimuli × 2 tasks × 18 subjects × 5 repetitions) for the Urdu dominant group/Cantonese-dominant group.

In the statistical analysis, raw data of response rate and reaction time were natural-logarithmically transformed to achieve better normality. On the base of sample size and the distribution of data, the linear mixed-effect model (LMM) was performed in R using the lme4 package (Bates et al., 2015), in the test field of individual response rate and reaction time. According to Baayen et al. (2008), LMM shows advantages in processing nested hierarchical data. The efficiency of the LMM model was examined by marginal *R*^2^ and conditional *R*^2^ using the MuMIn package (Bartoń, 2015) in R, which measures the variances explained by fixed or random effects (Nakagawa and Schielzeth, 2013; Zou et al., 2017). All *p*-values were corrected with Bonferroni adjustment for multi-comparisons.

For response rates and reaction time, initially a full model was run with fixed effects of subject groups (native Cantonese, Cantonese-dominant, and Urdu-dominant groups), experimental trails (segment-and-tone, and segment-or-tone), tone type (low-falling and low-rising), consonant type (/b, d, k, t/), and vowel type (/a, i/). However, the last three factors were removed from the models for both response rate and reaction time due to their insignificance. Once these factors were removed, response rate or reaction time served as the dependent variables in the LMM model, incorporating fixed effects of subject group and experimental task, as well as their interaction. For random effects, by-subject (56 levels) and by-item (16 levels) intercepts were included. In order to gain more insight into the individual variation of the data, the relationship between LDS and the task results (response rates and reaction time) was examined in a linear regression model, with LDS as an independent variable, and response rate or reaction time as a dependent field.

### Overall Results of Response Rates and Reaction Time

The statistical results of LMM are presented in Table 3. The LMM models showed efficiency with marginal *R*^2^ of 0.58 and conditional *R*^2^ of 0.78 for response rates and marginal *R*^2^ of 0.54 and conditional *R*^2^ of 0.73 for reaction time. It also reported the random effect of the by-subject intercept with a variance of 116.3 (*SD* = 10.79) and the by-item intercept with a variance of 65.32 (*SD* = 7.66) for response rates. For reaction time, the by-subject intercept showed a random effect with a variance of 0.02 (*SD* = 0.14) and the by-item intercept showed a variance of 0.09 (*SD* = 0.01).

**Table 3:** The results of LMM for response rates and reaction time.

**Fixed effects**	**Response rates**					**Reaction time**				
	**β**	***SE***	***df***	***t***	***p***	**β**	***SE***	***df***	***t***	***p***
*Interception*	157.67	4.81	129.55	32.82	<0.001	0.09	0.05	71.71	1.66	<0.001
*Subject group*	28.22	2.26	129.55	12.51	<0.001	0.43	0.03	71.42	16.89	<0.001
*Task type*	65.98	1.91	1,734	34.46	<0.001	0.89	0.01	8887	68.48	<0.001
*Subject group × Task type*	22.54	0.89	1,734	25.07	<0.001	0.2	0.06	8887	33.4	<0.001
**Random effects**	**Variance**	***SD***				**Variance**	***SD***			
*1/subject*	116.31	10.79				0.02	0.14			
*1/item*	65.32	7.66				0.09	0.01			
Marginal *R*^2^	0.58					0.54				
Conditional *R*^2^	0.78					0.73				

In terms of response rates (see in Table 3), according to LMM, there was a significant main effect in the subject group and in the task type. Moreover, LMM revealed an interaction between the subject group and task type, suggesting that native and non-native listeners performed differently across two ABX tasks. In terms of reaction time, LMM reported a significant main effect in the subject group and in the task type. Furthermore, the subject group significantly interacted with the task type in LMM. To answer the first research question, we analyze the data of the segment-and-tone and report the results in section The Task of Segment-and-Tone. Section The Task of Segment-or-Tone gives the results of the segment-or-tone and a comparison with that of segment-and-tone to answer the second research question.

### The Task of Segment-and-Tone

**Figure 3:**
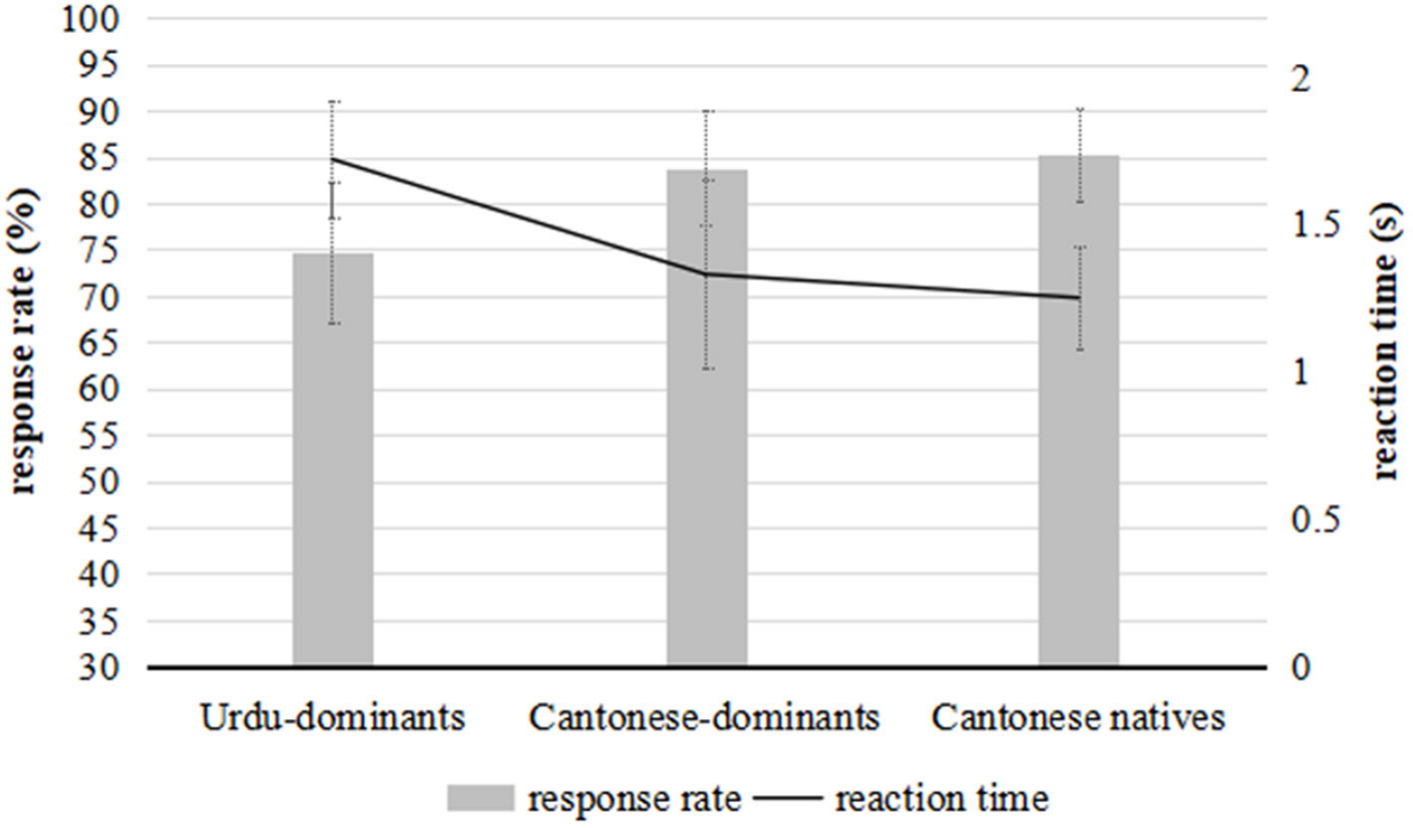
The mean accuracy/response rates (gray bars) and reaction time (dark line) for the bilinguals and Cantonese native speakers in the task of segment-and-tone. The minor y-axis shows the response time and the main y-axis illustrates the response rate. The error bars show 1/2 of *SD*.

The mean percentage of response rates and reaction times for the native and bilingual groups are exhibited in Figure 3. According to the *post-hoc* test, the Cantonese native speakers had slightly higher accuracy rates (*M* = 85.2%, *SD* = 10.11) and slightly shorter reaction time (*M* = 1.25 s, *SD* = 0.35) than the Cantonese-dominant bilinguals, who obtained a mean accuracy of 83.8% (*SD* = 12.51) with a mean reaction time of 1.33 s (*SD* = 0.64). However, the differences between the Cantonese native group and the Cantonese-dominant group were not statistically significant. This suggests that generally, the Cantonese-dominant bilinguals were able to accurately identify Cantonese stimuli as quickly as the Cantonese native speakers did in the segment-and-tone condition. In other words, when both segmental and tonal information were provided in the task, the Cantonese-dominant bilingual speakers could process tones as phonologically as native speakers did.

In comparison with the Cantonese-dominant bilinguals, the Urdu-dominant participants evidently needed (*z* = 0.14, *p* < 0.001) more time (*M* = 1.72s, *SD* = 0.96) to respond to the Cantonese stimuli, with significantly (*z* = 4.29, *p* < 0.001) lower accuracy rates/response rates (*M* = 74.6%, *SD* = 15.2). This indicated that Cantonese proficiency and experience facilitated the Cantonese-dominant bilinguals to perceive L2 stimuli more phonologically.

Generally, in the task of segment-and-tone, the speakers, whose maternal or dominant language is Cantonese, responded much more quickly and accurately than those who were dominant in Urdu. It was noted that as the mean accuracy of Urdu-dominants was far above chance level (50%), it was clear that the Urdu-dominant subjects were also able to process Cantonese stimuli phonologically, but in a much weaker way than the other two subject groups.

### The Task of Segment-or-Tone

**Figure 4:**
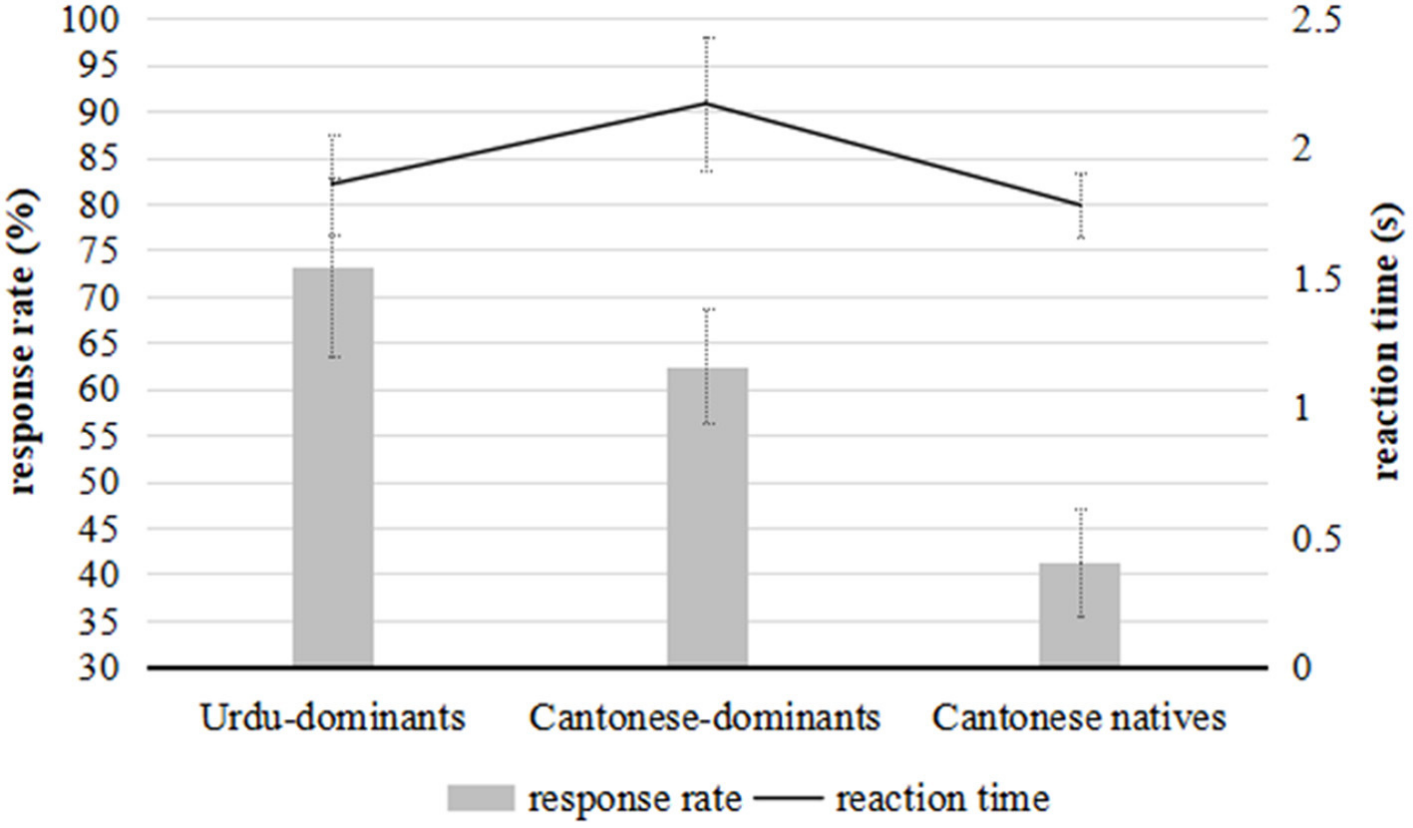
The mean accuracy/response rates (gray bars) and reaction time (dark line) for the bilinguals and Cantonese native speakers in the task of segment-or-tone. The minor y-axis shows the response time and the main y-axis illustrates the response rate. The error bars show 1/2 of *SD*.

The mean percentage of response rates and reaction times for the native and bilingual groups are exhibited in Figure 4. According to the *post-hoc* Tukey test, only 41.1% (*SD* = 11.62) Cantonese native speakers classified the stimuli along “segments,” far fewer (*z* = 3.33, *p* < 0.001) than the Cantonese-dominant bilinguals with a percentage of 62.5% (*SD* = 12.1) for “segments.” This illustrated that although the Cantonese-dominant bilinguals obtained a comparable performance with the native speakers in the task of segment-and-tone, they still performed significantly differently from the native speakers group. The Cantonese-dominant bilinguals paid more attention to the segmental dimension, while the native speakers were more sensitive to the tonal information.

Around 73.1% (*SD* = 19.35) of Urdu-dominant participants redistributed their attention more frequently (*z* = 3.19, *p* = 0.0093) to the segmental dimension when classifying Cantonese non-words than the Cantonese-dominant bilinguals did. Compared to the Cantonese-dominant bilinguals, the Urdu-dominant participants were evidently more attentive to the segmental information. Thus, the language dominance influenced how the bilinguals distributed their attentional resources.

Moreover, it is noteworthy that the Cantonese-dominant bilinguals (*M* = 2.17 s, *SD* = 0.81) responded much more slowly than both the native speakers (*M* = 1.78 s, *SD* = 0.25; *post-hoc*: *z* = 1.26, *p* = 0.0093) and the Urdu-dominants (*M* = 1.86 s, *SD* = 0.58, *z* = 1.81, *p* = 0.0186). The slow response for the Cantonese-dominants revealed a larger cognitive effort in making a decision on the stimuli. Urdu was their maternal language, and Cantonese was gradually becoming a strong language for them. On one hand, they did not feel able to ignore the attentional strategy (attentive to segments) in their L1, and on the other hand, they were not as immediately attentive to the tones as the Cantonese native speakers were. Hence, they needed much more time to resist their L1 strategy and produce a L2 attentional strategy.

No statistical difference was reported in reaction time between the Urdu-dominant bilinguals and the native speakers, suggesting that the Urdu-dominants were not necessarily subject to interference by the weaker language. Both groups responded immediately according to their native patterns of attention distribution. In the task of segment-or-tone, Cantonese native speakers distributed their attention mainly along tonal dimensions, while the bilinguals classified the stimuli mostly along segmental dimensions.

A comparison of the results of the two experimental tasks shows that the Cantonese natives (*z* = 1.27, *p* < 0.001), the Cantonese-dominant bilinguals (*z* = 2.43, *p* < 0.001), and the Urdu-dominant bilinguals (*z* = 2.87, *p* < 0.001) spent more time giving a response in the segment-or-tone task than in the segment-and-tone task, since the latter task was more cognitively demanding for the listeners.

## Discussion

In the segment-and-tone task, both accurate tonal and segmental information were provided, resulting in a comparatively low cognitive demand for the listeners. As predicted, most of the Cantonese native speakers as well as the bilinguals accurately identified the Cantonese stimuli, with accuracy ranging from 74.6 to 85.2%. In terms of the mean accuracy and reaction time, no statistical difference was detected between the Cantonese-dominant bilinguals and the Cantonese native speakers. In contrast, when one of the tonal and segmental dimensions was mismatched in the stimuli, as was the case in the task of segment-or-tone, all the subject groups, including the Cantonese native speakers, showed a much longer reaction time in making a decision than in the task of segment-and-tone. The more cognitively demanding task cost the listeners more time to process the tonal or segmental mismatch in the stimuli.

These results demonstrate that both native and bilingual speakers find it easy to make quick and accurate responses to the stimuli when there is no mismatch in the tonal or segmental dimension. This finding is in line with prior research on bilingualism (Antoniou et al., 2012; Amengual, 2016) showing that bilingual speakers are able to process Cantonese tones phonologically as native speakers do, when undertaking a comparatively less cognitively demanding perception task. Native and non-native speakers might perform comparably in a task with a low cognitive requirement, while the perceptual difference might be revealed by a comparatively high cognitively demanding task. For example, Amengual (2016) showed that Spanish-dominants and Catalan-dominants whose L1 is Spanish, could both categorically perceive the Catalan vowels in an categorical perceptual task where the speech sounds in the continuum varied along acoustic aspects of syllable duration and vowel formants. The reason is that the categorical perceptual task mainly examined the general auditory ability of listeners. However, when Amengual's bilinguals conducted a lexicon decision task, and had to attend to their long-memory of the lexicon system, a perceptual difficulty emerged for the early bilinguals. In addition, our research supports the findings of Strange (2011), indicating that when an easy perception task is conducted, it is possible for bilinguals to obtain a performance comparable with that of native speakers, because they have enough time and attentional resources to extract sufficient information to make an accurate decision.

With regard to the question as to how bilinguals distribute attention to tones and segments, the native and bilingual speakers, as discussed above, were able to rapidly make accurate responses in the task of segment-and-tone, since both tonal and segmental information were matched in the stimuli. As the task of segment-or-tone forced the listeners to respond along only one accurate phonetic dimension, the comparison between the results of the two tasks allows us to examine how the listeners distribute their attention toward tonal and segmental dimensions. The results showed that on average around 41.2% of the Cantonese native speakers classified the stimuli along the tonal dimension, resulting from their native attentional strategy. In Cantonese, tones convey lexical meanings in a syllable, so in order to extract the meanings carried by tones, Cantonese speakers are required to pay much of their attention to the tonal aspect.

This result coincides with the findings in Braun and Johnson (2011) and Zou et al. (2017), which studied the case of Mandarin, and demonstrated that tonal language speakers distribute their attention across both tonal and segmental dimensions in the perception of their native languages. The bilingual speakers in our task of segment-or-tone mainly classified the stimuli along segmental dimensions, with a mean response rate of around 66%. This illustrates that compared with the tonal native speakers, the bilingual speakers paid more attention to the segments than to the tones, which was similar to the performance of the Mandarin learners in Zou et al.'s study whose L1 was Dutch. Although the current study obtains the similar results with that of Zou et al. (2017), the current study recruited Cantonese-dominants and Urdu-dominants as the participants to explore the effect of language dominance on the selective attention of segments and suprasegments in Cantonese. The observations from the current study can make contribution to the field of second language acquisition and Chinese language teaching and learning.

In the task of segment-and-tone, no statistical difference was detected between the performance of the Cantonese-dominants and that of the Cantonese natives, while the Urdu-dominant bilinguals achieved significantly lower accuracy and required far more reaction time to make responses compared with the other two subject groups. Thus, the result supports the finding in Sebastián-Gallés and Soto-Faraco (1999) claiming that language dominance impacts the processing of L2 speeches, and the L2-dominant (Cantonese-dominant in the current study) speakers are able to perform in a more L2-like way, compared with the L1-dominants. This is because L2-dominant bilinguals are usually more proficient and experienced in their L2 language use, age of learning, and LOR (Flege and Fletcher, 1992; Piske et al., 2001).

In the task of segment-or-tone, 73.1% of the Urdu-dominants classified the stimuli according to segmental dimensions, and 62.5% of the Cantonese-dominants were attentive to the segmental information. This indicates that the Urdu-dominants had far more interference from their L1 attentional strategy, depending more on segments than the Cantonese-dominants did in processing Cantonese stimuli. In comparison, Zou et al.'s (2017) results showed that above 80% of Dutch-speaking beginner learners of Mandarin were attentive to segments, and nearly 70% of Dutch-speaking advanced learners of Mandarin classified the Mandarin stimuli along segmental dimensions. Therefore, the result of the Urdu-dominants in our study is closer to that of the advanced learners in Zou et al.'s study. Furthermore, the results of the Cantonese-dominants are different from tonal native speakers who focused mainly on tones, and from the beginner and advanced learners of Mandarin in Zou et al.'s study, who overtly paid attention to the segments. This supports the statement in Antoniou et al. (2012), that bilinguals should be treated as a unique and configured population, clearly different from a native one.

The results also showed that in the task of segment-or-tone the Cantonese-dominant group had a far longer reaction time in processing the mismatched tones and segments, than did the other two subject groups. This may be partly because although the Cantonese-dominants have mastered a certain awareness and knowledge of attentional strategy in Cantonese, it is not as automatic as it is for the Cantonese natives. Consequently, they are not able to respond as fast as native speakers in the task of segment-or-tone. In accommodating two language-specific attentional strategies at one time the Cantonese-dominants spend more time weighing up the strategies. In comparison, the Urdu-dominants are influenced more by their mother language, which dominates their language systems, so they activate attentional strategy to L1 very quickly, without necessarily spending extra time weighing between Urdu and Cantonese.

In considering how the experience of Urdu influences the bilinguals' processing of Cantonese stimuli, both the phonological impact and higher-order strategical influence were included in the current study. According to PAM-S, bilingual speakers may assimilate the low-rising Cantonese tone as Urdu question intonation, due to the similarity of rising pitch contours. Similarly, the Cantonese low-falling tone may be categorized as Urdu statement intonation, as it is comparable to a low-falling pitch tail and a descending pitch tendency at the end of a statement sentence (So and Best, 2011). PAM-L2 predicts that if listeners assimilate non-native sounds into different L1 categories, they will find it very easy to distinguish non-native sounds. Therefore, the phonological impact, if it indeed exists, would facilitate the processing of Cantonese for the bilinguals, and the perceptual differences shown in the task of segment-or-tone, would result from the impediment of the attentional strategy overtly used in the listeners' L1.

In Urdu, segments are used to classify a syllable, and it is suggested that such segment-dependent attentional strategy for non-tonal speakers would largely impede their processing of a tonal language (Zou et al., 2017). Therefore, bilinguals are not as sensitive as native speakers are when processing tonal information. The current study supports the prior findings on selective attention for native and non-native language listeners (Strange and Shafer, 2008; Steinhauer et al., 2009; Strange, 2011; Steinhauer, 2014; White et al., 2017; Zou et al., 2017), suggesting that non-native learners cannot develop a native-like selective perceptual routine, but their L2 processing can become increasingly automatic as they accumulate L2 experience.

Antoniou et al. (2012) suggested that bilinguals may have well-developed L1 and L2 systems, but that there is still an overlap between L1 and L2. We agree with these observations, since well-developed Urdu and Cantonese phonology systems enabled the bilinguals in our study to perceive phonologically the Cantonese stimuli in the task of segment-and-tone, whilst the overlap between languages allowed the bilinguals to co-activate different attentional strategies, as shown in the task of segment-or-tone. Due to the language overlap, the segment-dependent strategy the bilinguals used in Urdu system hindered the attention distribution of Cantonese segmental and tonal dimensions.

As discussed previously, the Cantonese-dominants performed similarly to the Cantonese natives in the task of segment-and-tone, and showed differences from the native speakers when exposed to the segment-or-tone task. Therefore, the Cantonese-dominants can be regarded as Cantonese speakers in the first task, and they shift to bilingual status in the second task. This suggests that even L2 dominant speakers cannot perform exactly like native speaker, in line with the previous findings of Sebastián-Gallés and Soto-Faraco (1999). Antoniou et al. (2012) found that English-dominant bilinguals whose L1 is Greek, behaved like monolingual speakers of English when perceiving a phonetic continuum of initial stops (in the categorical perception task). However, the English-dominants shifted their role to bilinguals when they were required to assimilate English and Greek initial stops (in the assimilation task). As outlined by Antoniou et al., the assimilation task required the listeners to refer to both their L1 and L2 phonology systems to make a goodness-of-fit rating between L1 and L2 phonetic contrasts. Antoniou et al. also indicated that PAM-L2 could explain how L1 phonology influenced L2 perceptual performance, but that it could not account for why the role of bilinguals shifted, to monolingual or bilingual, within different perceptual tasks. The flexible role outlined in Antoniou et al. (2012) and in the current study support the claims of Grosjean (2012a,b) language mode framework. Grosjean indicates that when bilinguals are exposed to only one language (language A), the stronger language (language A) will be highly activated, and the weaker language (language B), will be slightly or hardly activated, which generates a monolingual mode. When the listeners are provided with both languages (language A and B), the two languages will be activated to a large degree, but the weaker language (language B) will be slightly less activated since it is not dominant for the bilingual speaker, resulting in a bilingual mode. In fact, the language mode in Grosjean's model concerns the flexible roles that bilinguals play in sound processing. We define the flexibility as the ability of a bilingual speaker to shift roles between bilingual and monolingual mode under different tasks, the “perceptual flexibility” proposed by Antoniou et al. (2012).

Grosjean (2012b) had a definition of “monolingual mode,” requiring bilinguals to have a very high proficiency in L2, and the laboratory setting does not allow any activation of another language for bilinguals. However, as Grosjean suggested, “putting bilingual participants in a monolingual mode in a research project is difficult.” There have been other research attempts to control a monolingual mode, such as monolingual experimental materials, monolingual testing scripts, etc., for example by Antoniou et al. (2012) and Sebastián-Gallés and Soto-Faraco (1999).

For the purposes of the current study, the experimental stimuli were produced in Cantonese, the facilitators were Cantonese native speakers, and only Cantonese scripts were offered to the listeners, creating a monolingual mode for the bilinguals. In the task of segment-and-tone, the monolingual mode allowed the Cantonese system (the stronger language) to be activated for the Cantonese-dominants, who are highly experienced and proficient in Cantonese, which is why they performed comparably to the Cantonese native speakers. However, for the task of segment-or-tone, the Cantonese-dominants performed quite differently from the Cantonese native speakers, even though they were highly experienced and only the Cantonese mode was provided in the experiment. One possible explanation may be that the task of segment-or-tone not only examines whether the bilinguals have established Cantonese phonetic categories, but also provides links to their executive functions, which the bilinguals can use to process the tonal and segmental mismatches in the stimuli.

The executive function of selective attention correlates with the selective perceptual routine, suggested by Strange and Shafer (2008), and Strange (2011). According to Grosjean (1998), for a bilingual speaker, the stronger and weaker languages are domain-specific and have dynamic systems instead of static and unchanged ones. A weaker language can become dominant for the bilingual speaker, when they are exposed to an unknown or unfamiliar linguistic context, language domain, or experimental task, and vice versa for a stronger language to change into a weaker one. The earliest systematic Cantonese learning for the bilingual middle school students started in the classroom. Teachers may teach explicit knowledge, such as how to distinguish tones, use vocabulary, and organize sentences grammatically. However, they often neglect tacit knowledge teaching, such as attentional strategy and meta-cognitive knowledge, important for students to develop a native-like attentional strategy. In the executive domain of language processing, native speakers have already developed a mature and automatic strategy to deal with a task although they are usually unaware of it. However, the bilinguals, who never pay attention to it or have not developed a Cantonese-specific attentional strategy, will naturally refer to their native language system, and make responses based on their L1.

With respect to Chinese language learning and teaching, the current study suggests that tonal perceptual training is still an essential part as Urdu-Cantonese bilinguals cannot perceive Cantonese tones like native speakers. Apart from formal classroom teaching, online perception training with L2 syllables, disyllabic words, and conversational materials can be adopted to enhance non-native learners' communicative ability. Also, the “one-to-one” mode provides flexibility in perceptual training, which allows the training procedure to be adjusted based on individual learners' backgrounds and language proficiency.

## Conclusions

To examine how Urdu and Cantonese dominant bilingual speakers distribute their attention when processing Cantonese tones and segments, a cognitively demanding task was conducted. The results showed that the bilinguals, especially the Cantonese-dominants, were able to process Cantonese tones in a phonological way when both segmental and tonal information was accurately matched (the segment-and-tone task). However, they were impeded by Urdu attentional strategy when tonal and segmental dimensions were mismatched. The results supported Strange (2011) research as well as that of Strange and Shafer (2008), suggesting that non-native listeners can obtain a more automatic selective perceptual routine in L2 as they gain L2 experience. However, even L2 dominant bilinguals still cannot completely overcome the interference of the attentional strategy of their L1.

Moreover, the Cantonese-dominants performed in a monolingual way in the first task (segment-and-tone) and performed like bilinguals in the second task (segment-or-tone). This finding coincides with the research of Antoniou et al. (2012), proposing that highly experienced bilinguals show a perceptual flexibility when conducting different tasks. PAM-L2 cannot account for such flexibility, but it can be explained by the framework of Grosjean's language mode. It also suggests that the bilinguals should be treated as a unique language group instead of being regarded as native speakers of both languages. Furthermore, being more dominant in Cantonese clearly enabled the Cantonese-dominants to gain a more Cantonese-like performance compared with the Urdu-dominants. The relationship between the listeners' perceptual performances and their degree of language dominance indicates that the methodology of PAM-L2 can predict bilinguals' performances.

## Data Availability Statement

The original contributions presented in the study are included in the article/supplementary material, further inquiries can be directed to the corresponding author/s.

## Ethics Statement

The studies involving human participants were reviewed and approved by Hong Kong Polytechnic University. Written informed consent to participate in this study was provided by the participants' legal guardian/next of kin.

## Author Contributions

Both authors listed have made a substantial, direct and intellectual contribution to the work, and approved it for publication.

## Conflict of Interest

The authors declare that the research was conducted in the absence of any commercial or financial relationships that could be construed as a potential conflict of interest.

## Publisher's Note

All claims expressed in this article are solely those of the authors and do not necessarily represent those of their affiliated organizations, or those of the publisher, the editors and the reviewers. Any product that may be evaluated in this article, or claim that may be made by its manufacturer, is not guaranteed or endorsed by the publisher.
